# Assessment of the Potential for Producing Geopolymer-Based Granulates as a Substitute for Natural Aggregates

**DOI:** 10.3390/ma18235275

**Published:** 2025-11-21

**Authors:** Magdalena Cempa, Jerzy Korol, Agnieszka Klupa

**Affiliations:** 1 Department of Environmental Analysis and Circular Economy Technologies, Central Mining Institute–National Research Institute, Pl. Gwarków 1, 40-166 Katowice, Poland; aklupa@gig.eu; 2 Department of Mechanical Research and Materials Engineering, Central Mining Institute–National Research Institute, Pl. Gwarków 1, 40-166 Katowice, Poland

**Keywords:** aggregate, coal fly ash, composite regrind, geopolymer, granulate

## Abstract

This study presents the development and evaluation of a technology for producing geopolymer-based granulates, which act as sustainable substitutes for natural aggregates by utilizing waste materials. The technology is demonstrated to be energy-efficient compared to other manufactured aggregate processes (such as sintering), as it relies on a cold-bonding process and achieves self-hardening at room temperature. The granulation of geopolymer materials using an intensive counter-current mixer represents an innovative solution in the field of producing substitutes for natural aggregates. Coal fly ash (CFA) was used as the primary aluminosilicate precursor, with composite regrind from decommissioned wind turbine blades (CR) and steelmaking dust (SD) tested as additives. Reactive solids and alkaline activator liquids were mixed and granulated in a single operation using an intensive counter-current mixer; moistening and surface powdering were applied to improve granule sphericity. The granules were cold-cured at room temperature and characterized after 28 days by grain size distribution, crushing resistance, water absorption, abrasion (micro-Deval), SEM/EDS and leaching tests. The results indicate that the additives significantly improved the mechanical performance: PM + PK granules reached crushing strengths > 6 MPa, while CFA + SD granules reached > 11 MPa, exceeding many commercial lightweight aggregates (such as LECA or Lytag), as detailed in the paper. The CFA + CR granulates exhibited a compact microstructure and the effective immobilization of several heavy metals, whereas the CFA + DS samples demonstrated the excessive leaching of Cr, Pb and Mo. The process achieved a high solid-to-liquid ratio (>2.0), reducing activator consumption. Composite regrind is recommended as a promising additive.

## 1. Introduction

Crushed natural rocks and stones serve as the main coarse aggregates in concrete, with variants such as limestone, basalt and gravel chosen according to regional geology. However, increasing construction activity and the rapid exhaustion of these resources threaten the sustainability of the sector [1,2,3]. Three technical strategies have been suggested to overcome these challenges: recovering aggregates from demolished concrete [4,5,6,7], using coarse waste materials (bottom ash or steel slag) directly as aggregates [8,9], and producing manufactured aggregates from industrial waste or by-products [10,11].

Geopolymers, formed through the alkali activation of aluminosilicate precursors, can replace natural aggregates and may offer beneficial physical, chemical and mechanical properties comparable to those of ordinary Portland cement [12]. Currently, researchers increasingly emphasize waste material reuse and recycling, especially within the field of geopolymers [13,14]. Geopolymers typically require feedstocks rich in silica and alumina (together usually exceeding 50% of the total), since these oxides form the structural backbone of the material. Consequently, choosing the appropriate aluminosilicate precursors is essential when producing geopolymers intended as synthetic aggregates. A range of aluminosilicate sources, including kaolin [15], metakaolin [16], fly ash [17], slag [18], red mud [19], rice husk ash [20] and volcanic ash [21], have been widely investigated for this purpose. Other potential materials, such as granite [21], waste glass powder [22,23] and quarry tailings [24], have also been actively researched as geopolymer precursors.

Apart from selecting appropriate precursors for geopolymer-based artificial aggregates, choosing suitable mixture proportions that effectively drive the geopolymerization process is also crucial. The key parameters include the alkaline solution concentration, the alkali activator ratio and the solid–liquid ratio between the aluminosilicate feedstock and the alkaline activator, which are often expressed as molar ratios. Both potassium hydroxide (KOH) and sodium hydroxide (NaOH) are often used as alkali solutions in the synthesis of geopolymers, generally in combination with K_2_Si_2_O_3_ or Na_2_SiO_3_ [25].

In addition to adjusting the liquid composition of the geopolymer-based artificial aggregates, the solid–liquid (S/L) ratio is a key factor that affects the properties of the obtained materials. The S/L ratio also strongly influences the chemical environment during geopolymerization. In general, increasing the S/L ratio (i.e., increasing the solid content) accelerates geopolymerization, as more Si and Al become available to form geopolymer bonds. An appropriate S/L ratio is therefore essential to promote effective geopolymerization and produce high-performance geopolymer-based artificial aggregates. However, if the S/L ratio becomes too high, there will be insufficient liquid for the reaction, which can result in poorly formed bonds [26,27,28].

Geopolymer production generally involves three stages: (i) mixing reactive solids and activator liquids, (ii) shaping (pelletization, molding, crushing or hand shaping), and (iii) curing (cold bonding, sintering and autoclaving) [29].

Cold bonding is a hardening technique for manufactured aggregates that involves air drying in an enclosed space for periods such as one, three or seven days at relatively low temperatures (under 100 °C). Cold-bonded aggregates are often cured with water or covered with plastic sheets until they gain adequate strength. Given that it does not require additional heating, cold bonding is an energy-efficient method commonly used with cementitious materials such as coal fly ash, iron ore tailings and granulated blast-furnace slag; the aggregate strength is primarily developed through cement hydration and/or pozzolanic reactions [30]. Gesoglu et al. [31] noted that producing lightweight fly ash aggregates via cold bonding consumes less energy and is more cost-effective than sintering.

The reported physical and mechanical data for the obtained geopolymer-based artificial aggregates reveal a wide range of physical and mechanical properties depending on the raw materials and processing (Table 1).

With the progressive depletion of natural resources and growing environmental constraints, the construction sector needs to develop alternative aggregate materials. The geopolymer granules developed in this study respond to these challenges by using industrial waste (fly ash, steel mill dust, and composite regrind) as secondary raw materials. This solution combines the circular economy aspect with the real potential to replace natural aggregates in construction and insulation applications.

Unlike commercial lightweight aggregates, which require thermal sintering processes, the proposed technology allows comparable mechanical parameters to be obtained under room temperature curing conditions, which significantly reduces the energy consumption of the process.

The growing pressure for sustainable development in the construction sector is motivated not only by environmental considerations but also by economic ones. The rising costs of extracting and transporting natural aggregates, combined with increasing fees for industrial waste storage, are a key driver for the search for technologies that enable their valorization. The aim of the developed technology is to transform waste, which has so far been a financial burden, into a fully fledged market product in the form of geopolymer granules that can replace natural aggregates.

Although the processes of alkaline activation, granulation, and low-temperature curing are well known, this study introduces a new dimension by: (i) using wind turbine blade grinding (PK) and steel mill dust (PS) as functional additives in geopolymer granules, (ii) applying a simultaneous activation and granulation step in an intensive counter-rotating mixer at S/L > 2.0, which reduces activator consumption, and (iii) simultaneously evaluating strength and leachability against environmental limits.

Therefore, the aim of this study was not only to develop a method for industrial waste disposal, but above all to demonstrate that it can serve as a basis for a ‘sustainable substitute’ for natural aggregates. In the context of this study, a ‘sustainable substitute’ is defined as a material that (1) is based on waste raw materials, (2) is produced in a low-energy-intensive process, and (3) has final physical and mechanical properties at least equal to, and optimally superior to, commercially available natural or sintered aggregates.

## 2. Materials and Methods

### 2.1. Materials

**Figure 1:**
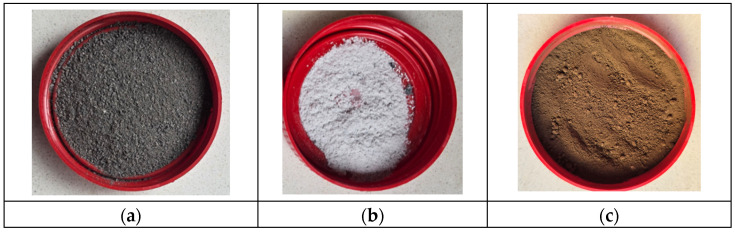
Photographs of materials: (**a**) coal fly ash (CFA), (**b**) composite regrind (CR), and (**c**) steelmaking dust (SD) used as additives in the production of geopolymer granules. Note: the photos are illustrative of the bulk materials’ appearance; detailed particle size characterization is provided in Tables 6 and 7 and Figures 5 and 6.

Coal fly ash from the combustion of hard coal in a fluidized coal boiler was used as the main precursor material to produce a geopolymer granulate (CFA). Steelmaking dust (SD) and composite regrind (CR) were used as additives for the geopolymer granule production (Figure 1). The steelmaking dust originated from waste gas dedusting during metallurgical processes. The composite regrind was obtained from wind turbine blades that had been taken out of service.

A composite regrind from decommissioned wind turbine blades is typically produced by mechanical grinding or milling, resulting in fine powders or shredded fibers. These materials are then used as fillers or reinforcements in new composites, such as polypropylene or geopolymer matrices, or as additives in cementitious materials. Mechanical recycling is the most cost-effective and widely adopted method, although it often leads to some reduction in mechanical properties compared to the original materials. However, optimized processes such as solid-state shear milling and surface modification can significantly increase the strength and modulus of the resulting composites, making them suitable for high-performance applications in construction and infrastructure [35,41,42,43,44,45].

### 2.2. Chemical Reagents

A binary combination of a sodium hydroxide solution (NaOH) and a sodium silicate solution (Na_2_SiO_3_) was used as the alkaline activator. Sodium hydroxide was supplied by the local company Avantor/POCH^®^ (Gliwice, Poland) in the form of white pellets. The sodium hydroxide was characterized by a molar mass equal to 40 g/mol and a purity not less than 99.4%. The sodium silicate solution, also known as water glass or liquid glass, was obtained from Chempur^®^ (Piekary Śląskie, Poland), a manufacturing company, in the form of a thick liquid. According to the producer, the density of the liquid sodium solution at 20 °C was 1.47 g/cm^3^, and its silica molar ratio (SiO_2_/Na_2_O) was 2.51.

### 2.3. Experimental Procedure

Various mixtures were prepared as part of the study, as presented in Table 2. It should be emphasized that the presented experiment plan (Table 2) was not a systematic parametric analysis, but rather a process of technological optimization. The goal was to develop a complete method for producing granules with the desired properties. The modifications introduced to the process, such as additional moisturizing (M) or powdering (P), were aimed at improving the sphericity and grain size of the product. At the same time, they forced the correction of other parameters, especially the S/L ratio, in order to maintain the appropriate workability of the mixture during granulation. Therefore, the individual recipes, compared later in this paper, differ in several variables at the same time, reflecting the iterative pursuit of an optimized final product.

**Figure 2:**
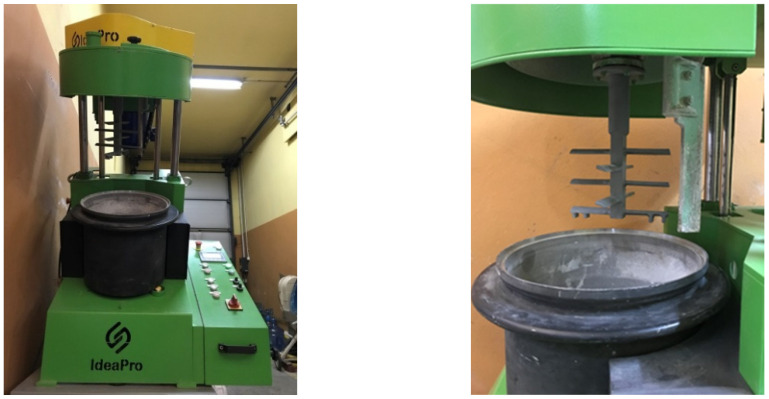
Production of geopolymer-based granulates in an intensive counter-current mixer.

The reactive solids and activator liquids were mixed and shaped into granules by means of an intensive counter-current mixer with a nominal capacity of 30 L. Granulation, i.e., the controlled agglomeration of highly dispersed raw materials, is a technique aimed at either preparing fine-grained substances for further technological operations or giving them the shape and properties characteristic of the final product [46,47]. During the processing of the tested materials, the mixer was equipped with a star-type paddle agitator (Figure 2). The design of this agitator enabled the disintegration and fragmentation of the agglomerated raw materials. The high linear speed of the agitator, which was about 30 m/s, allowed this stage to be carried out by operating the bowl and agitator in counter-current mode. The next stage of the process was homogenization to ensure a uniform composition of the mixture throughout the entire volume of the batch as well as its granulation in the final stage. Moistening and powdering of the material surface was applied during granulation to obtain granules with shapes as close as possible to spherical.

It is worth noting that the activation and granulation process was carried out in a single stage in an intensive counter-current mixer, which made it possible to obtain a high solid-to-liquid ratio (S/L > 2.0) during curing at room temperature.

The fresh geopolymer mixture was spread onto metal trays and left under room conditions. After 28 days, selected samples were subjected to a series of mechanical, physical and chemical tests.

### 2.4. Methods of Material Characterization

The main chemical components of the input materials and the geopolymer-based granulates were determined via wavelength dispersive X-ray fluorescence spectroscopy (WDXRF) by means of a ZSX PRIMUS II analyzer (Rigaku, Tokyo, Japan) equipped with a 4 kW X-ray Rh tube.

The trace chemical components of the input materials and the geopolymer-based granulates as well as the water leaching extract were determined via inductively coupled plasma optical emission spectroscopy (ICP–OES) using an Elmer Optima 5300 analyzer (PerkinElmer, Waltham, MA, USA). The analysis of the solid materials was performed after they were mineralized in a mixture of acids (30% HCl, 65% HNO_3,_ water) prepared according to the relevant standard. The water extracts were prepared at a ratio of 1 kg:10 L with accuracies of ±0.01 g and 0.05 mL, respectively [48]. The water extract pH was determined via the potentiometric method per standard [49].

Diffractometric tests were performed using the powder method (DSH) in Bragg–Brentano geometry with a Bruker D8 DISCOVER diffractometer (Bruker Corp., Billerica, MA, USA), CuKα radiation, Ni filter, and LYNXEYE_XE detector (Bruker Corp., Billerica, MA, USA). The mineral composition was determined and calculated based on standards licensed in PDF-5+ 2024 RDB ICDD (International Centre for Diffraction Data) and databases: ICSD (Inorganic Crystal Structure Database) and NIST (National Institute of Standard and Technology). DIFFRAC v.5.2 and TOPAS v.4.2 software from Bruker AXS was used for recording and diagnostics. Quantitative calculations of crystalline phases and amorphous substances were performed based on the Rietveld method, using ZnO or Al_2_O_3_ standards, depending on the sample matrix.

Analyses of grain morphology and chemical composition in the microareas were carried out by scanning electron microscopy (SEM) and X-ray energy dispersion spectroscopy (EDS) using an SU3500 SEM (Hitachi, Ltd., Tokio, Japan) operating in conjunction with an UltraDry EDS Detector (Thermo Fisher Scientific Inc., Waltham, MA, USA) under the following conditions: 15 keV acceleration voltage, BSE detector, magnification from 100× to 1000×. The images were taken after spraying the samples with gold.

Grain size and shape analysis via the optical method with image analysis using a G3S-ID analyzer (Malvern Panalytical Ltd., Malvern, UK) was carried out under the following conditions: air as the dispersion medium; dispersion pressure of 4.0 bar; ×20, ×10 and ×5 lenses; diascopic light.

**Figure 3:**
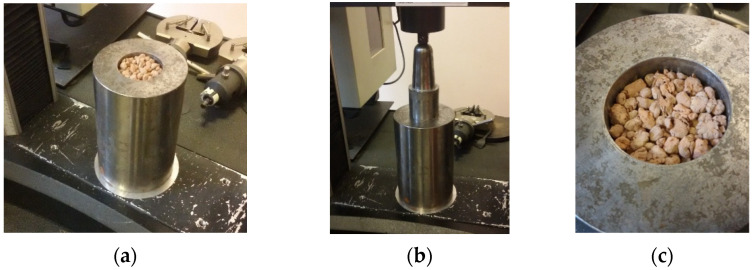
Crushing resistance testing of geopolymer-based granulates: (**a**) granulated sample in a steel cylinder–before testing, (**b**) granulate compression, (**c**) granulate after compression.

The crushing resistance of the geopolymer-based granulates was determined according to the standard [50]. Per the standard, crushing resistance tests can be performed for aggregates with a grain size of 4 mm to 22 mm and a bulk density greater than 150 kg/m^3^. Therefore, fractions below 4 mm and above 10 mm were removed from the prepared samples for testing individual granulates to homogenize the samples for comparative testing. Individual large granules in the tested material volume could distort the reading of the force required for the piston to penetrate the tested material in accordance with the standard. The tested granules were subjected to mechanical testing consisting of uniaxial compression of the samples in a steel cylinder in a strength testing machine with a maximum force of 30 kN (Figure 3). The crushing resistance (SC) of individual aggregates was calculated via the following equation:

(1) $$SC=\frac{L+F}{A}\;N/{mm}^{2}$$

where SC is the crushing resistance (MPa), L is the force exerted by the piston in newtons(N), F is the force required to depress the piston (N), and A is the piston area (mm^2^).

The water absorption testing of the geopolymer-based granulates was performed according to standard [51]. Granules with grain sizes ranging from 4 mm to 8 mm were placed in baskets and flooded with water to a height of approximately 5 cm above the tested sample level. The tested material was then left in the water until the samples were completely saturated (24 h). After this time, the granules were removed, wiped with a damp absorbent cloth, and weighed (m_1_). The next step involved drying to a constant mass (at 105 ± 5 °C). After removal from the dryer, the samples were weighed (m).

The aggregate water absorption was determined via the following formula:

(2) $$W_{A}=\frac{m_{1}-m}{m}\cdot 100\%\;$$

where m is the mass of the sample dried to a constant mass and m_1_ is the mass of the sample completely saturated with water.

The aggregate abrasion resistance was determined via the micro-Deval wet method per standard [52]. Granules with grain sizes ranging from 4 mm to 8 mm were used for the tests. The samples were rinsed in water and dried to a constant weight at 105 ± 5 °C. Samples weighing 500 g each were placed in the device container, and 2.8 dm^3^ of water were added. The device rotated the containers at a speed of 100 rpm. The set number of revolutions was 12,000 ± 10 revolutions. After the device was stopped, the containers were opened, and their contents were transferred to a protective sieve with a mesh size of 10 mm. The protective sieve was placed on another sieve with a mesh size of 1.6 mm. The material and steel balls on the sieves were rinsed with a stream of clean water. The balls were carefully separated from the material on the protective sieve. The remaining mineral material was transferred to a sieve with a mesh size of 1.6 mm and rinsed with water. The material remaining on the sieve was transferred to a tray, dried at 105 ± 5 °C and weighed. The micro-Deval coefficient was calculated via the following formula:

(3) $$Mde=\frac{500-m}{5}$$

where Mde is the micro-Deval coefficient, and m is the mass of the fraction retained on the 1.6 mm sieve [g].

Size distribution was determined via wet sieving methods according to standard [53] using a laboratory vibrating screen and standardized laboratory sieves (diameter—200 mm; height—50 mm; mesh sizes 1.6 mm, 2 mm, 4 mm, 8 mm and 16 mm) produced in accordance with the standard.

## 3. Results and Discussion

### 3.1. Characterization of the Materials Used as the Main Precursor for Producing the Geopolymer-Based Granulate

The main components of the coal fly ash (CFA) were silicon (SiO_2_, 30.0 wt%) and calcium (CaO, 20.9 wt%). The contents of aluminum (Al_2_O_3_) and iron (Fe_2_O_3_) were 22.2 wt% and 5.4 wt%, respectively (Table 3). The total content of SiO_2_, Al_2_O_3_ and Fe_2_O_3_ was 57.6 wt%. The tested ash belonged to the high-calcium ash group [54,55].

**Figure 4:**
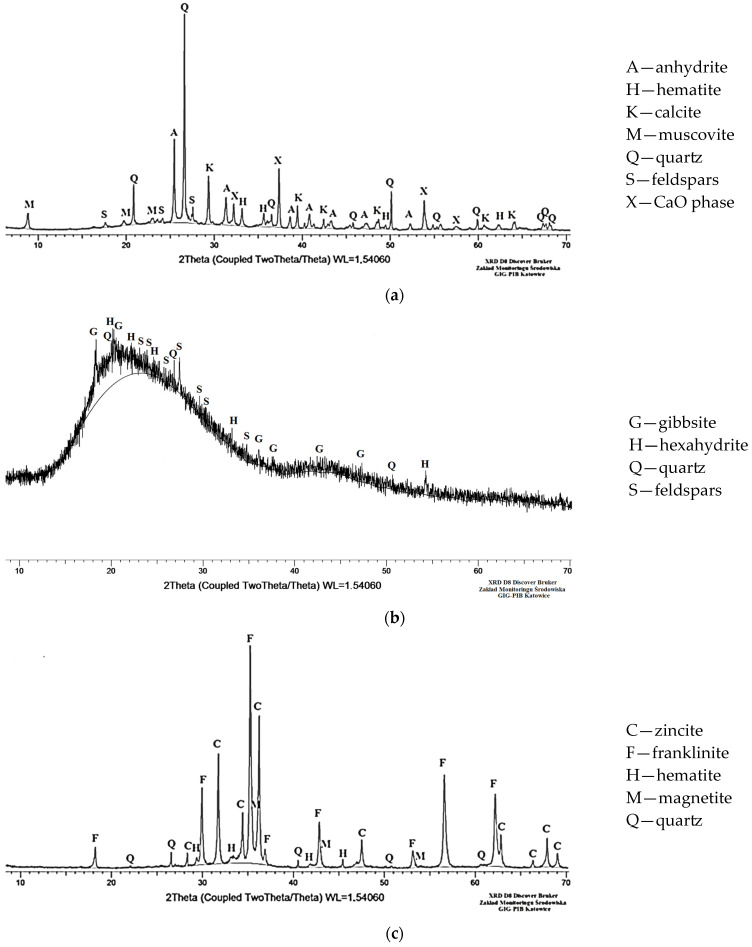
Mineral composition (via XRD) of the main precursor used for geopolymer-based granulate production: (**a**) coal fly ash (CFA), (**b**) composite regrind (CR), and (**c**) steelmaking dust (SD).

**Figure 5:**
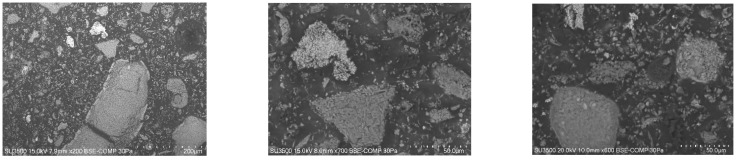
SEM images with grains of the coal fly ash (from the combustion of hard coal in a fluidized coal boiler, CFA) used as the main precursor for geopolymer-based granulate production.

**Figure 6:**
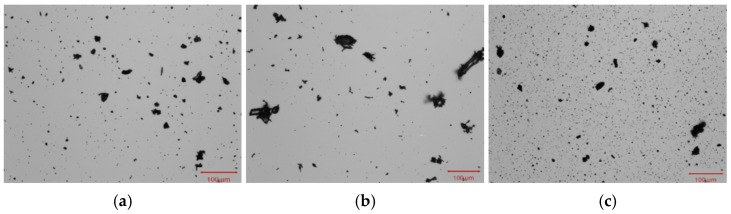
Photos of selected grains: (**a**) coal fly ash (CFA), (**b**) composite regrind (CR), and (**c**) steelmaking dust (SD) used as the main precursor in the production of geopolymer granules based on ash.

The ash also contained metals that are known for environmental toxicity and adverse effects on human health, such as arsenic, cadmium, lead, chromium, nickel, copper, zinc, tin, cobalt, molybdenum, antimony, barium, manganese and selenium [56,57]. Among the metals listed, the highest contents were identified for Zn (451 mg/kg), Ba (317 mg/kg) and Mn (304 mg/kg). The lead content did not exceed 200 mg/kg, the chromium and copper contents did not exceed 70 mg/kg, the nickel and cobalt contents did not exceed 40 mg/kg, and the contents of the remaining elements were less than 4 mg/kg. The total content of the analyzed elements was 1439 mg/kg (Table 4 and Table 5). The analyzed coal fly ash consisted mainly of mineral phases such as quartz (31.5%) and anhydrite (14.0%). Amorphous substances accounted for 35.5% (Figure 4). The ash grains were irregular in shape. The percentage of grains with a size (CE parameter) less than 10 µm was 96.2%. The percentage of grains below 1 µm was 14.0%. The grain size did not exceed 150 µm (Figure 5 and Figure 6, Table 6 and Table 7). The maximum grain sizes (CE parameter) for the composite regrind and steelmaking dust were 450 µm and 400 µm, respectively (Table 5).

The main chemical components of the composite regrind (CR) were carbon (C, 34.1 wt%) and silicon (SiO_2_, 30.7 wt%). Like the coal fly ash, this material was characterized by a high calcium content (CaO, 19.6 wt%). The sum of SiO_2_ and Al_2_O_3_ was 39.8 wt%. The contents of the other elements did not exceed 3 wt%. The chemical composition of the steelmaking dust (SD) was different from that of the coal fly ash. The main components were iron (Fe_2_O_3_, 37.2 wt%) and zinc (ZnO, 36.2 wt%). The sum of SiO_2_ and Al_2_O_3_ was 2.8 wt% (Table 3).

The amorphous phase in the composite regrind accounted for 84.0 wt% of the material. The identified crystalline components included gibbsite, hexahydrite, quartz and feldspar. The main mineral components in the steelmaking dust were franklinite (34.0 wt%) and zincite (24.0 wt%), while others included magnetite (14.0 wt%), quartz (4.5 wt%) and hematite (4.0 wt%). The amorphous phase constituted 17.5 wt% of the steelmaking dust (Figure 4).

The total contents of metals such as arsenic, cadmium, lead, chromium, nickel, copper, zinc, tin, cobalt, molybdenum, antimony, barium, manganese and selenium were 193 mg/kg and 326 g/kg, respectively, for the composite regrind and steelmaking dust. The high metal content in the steelmaking dust was primarily due to the high levels of zinc (291 g/kg) as well as manganese (18.9 g/kg), lead (9.5 g/kg) and chromium (3.6 g/kg). The total content of the remaining elements (arsenic, cadmium, nickel, copper, tin, cobalt, molybdenum, antimony barium, and selenium) was 3375 mg/kg.

Analyzing the input materials for the leachability of the abovementioned elements revealed that the coal fly ash (CFA) and composite regrind (CR) were moderately stable, whereas the steelmaking dust (SD) was characterized by a high mobility of selected elements. The coal fly ash mainly leached barium (3.46 mg/kg), antimony (0.70 mg/kg) and molybdenum (0.51 mg/kg). The composite regrind primarily leached cobalt (2.35 mg/kg) and molybdenum (0.85 mg/kg), as well as manganese (0.46 mg/kg), zinc (0.22 mg/kg) and barium (0.08 mg/kg). Despite the high zinc content, molybdenum was the main element leached from the steelmaking dust (91 mg/kg), accounting for 33% of all the leached elements and 33% of the total molybdenum content. In addition, lead (133 mg/kg), chromium (25.3 mg/kg), barium (12.3 mg/kg), zinc (8.1 mg/kg), selenium (1.4 mg/kg), antimony (0.25 mg/kg) and copper (0.11 mg/kg) were leached as well.

### 3.2. Grain Composition and Granulate Shape

**Figure 7:**
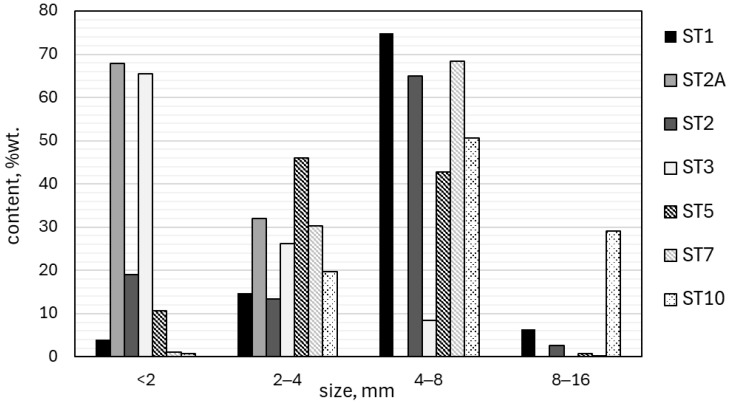
Grain size composition of geopolymer-based granulate.

Clear differences between individual samples can be observed in the analysis of the geopolymer granule grain composition (Figure 7). The grain fraction with a size of 4–8 mm was dominant in samples ST1 and ST2, accounting for 74.9% and 64.9% of the total mass, respectively. The opposite trend was observed in samples ST2A and ST3, where the grain fraction below 2 mm was clearly dominant, reaching 67.9% and 65.4% of the total mass, respectively, whereas the share of grains above 4 mm did not exceed 8.4%. 

**Figure 8:**
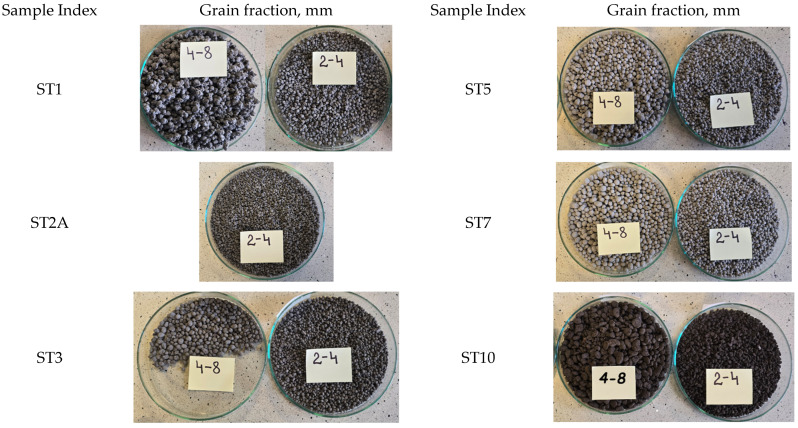
Photos of the produced geopolymer-based granulate.

In the case of samples ST5 and ST7 (based on coal fly ash and composite regrind), 2–8 mm was the main fraction size; specifically, in the case of sample ST5, the 2–4 mm and 4–6 mm fractions were similar and amounted to 46.0% and 42.7% of the total mass, respectively, whereas the 4–8 mm fraction was dominant in the case of sample ST7 (68.3% of the total mass). The most diverse grain size distribution was found in sample ST10, which contained all the analyzed fractions, including the largest grains of 8–16 mm, which accounted for 29% of the total mass. Figure 8 presents photographs of the granules in the 2–4 mm and 4–8 mm grain size fractions.

Fine granules with a moist surface formed during the homogenization stage of the ST1 sample components, which conjoined during the maturation stage, creating agglomerates with uneven surfaces. In experiments ST2 and ST2A, the solid-to-liquid ratio was increased to 2.00 and 1.90, respectively (Table 2). The application of staged moistening enabled the formation of spherical granules larger than 4 mm in experiment ST2. In experiment ST3, the use of surface powdering made it possible to produce fine (2–4 mm) spherical granules.

### 3.3. Mechanical and Physical Properties of the Produced Geopolymer-Based Granulates

**Figure 9:**
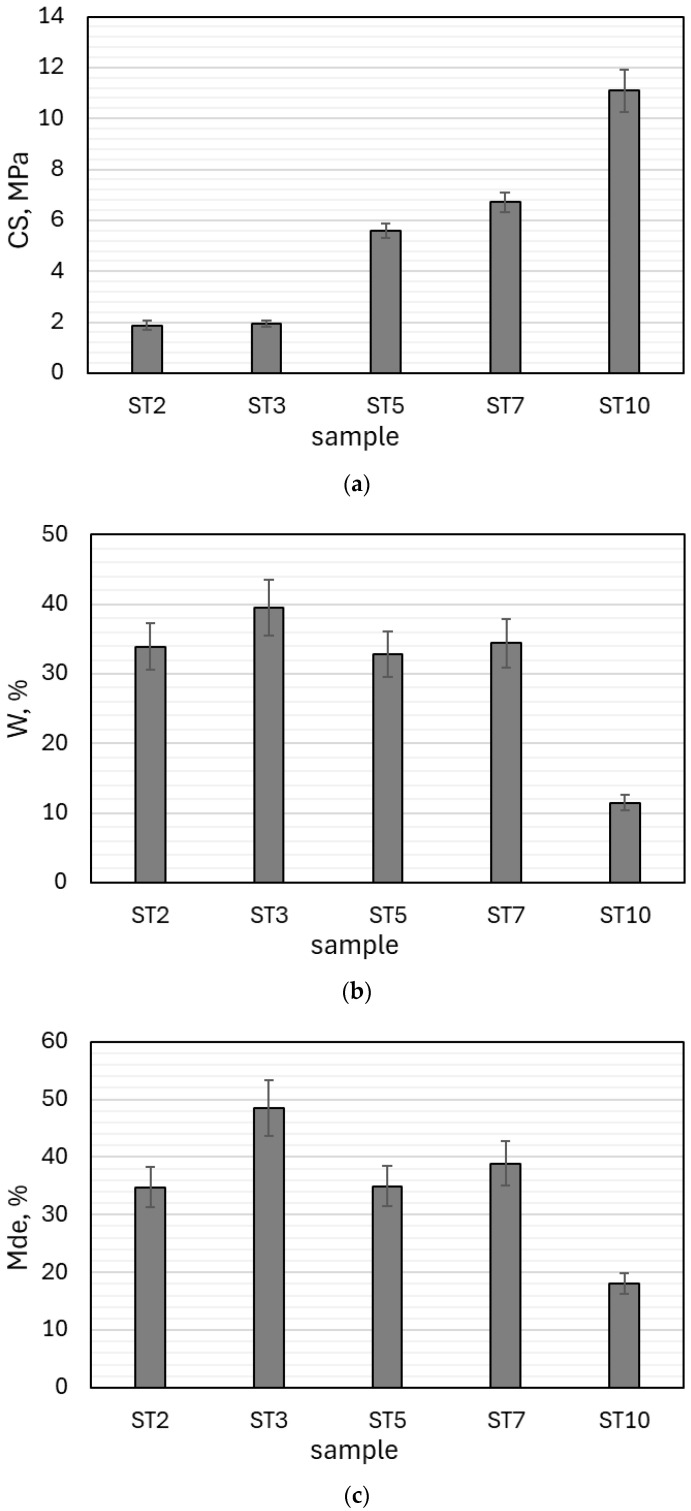
Results of mechanical and physical property testing for geopolymer-based granulates: (**a**) crushing resistance, (**b**) water absorption, and (**c**) abrasion resistance.

The results of crushing resistance, water absorption and abrasion resistance tests performed for the geopolymer-based granulates after 28 days of curing are depicted in Figure 9. The use of additives such as composite regrind (CR) and steelmaking dust (SD) had a significant effect on the mechanical properties of the geopolymer granulates. The crushing resistance of the (CFA) geopolymer granulate (based on fly ash) did not exceed 2 MPa. The use of composite regrind as an additive improved the mechanical properties of the material. In the case of sample ST5, there was a 2.9-fold increase in value (CS = 5.6 MPa), and in the case of sample ST7 (where powdering was used in addition to moistening), a 3.4-fold increase in value was recorded (CS = 6.7 MPa). In the case of test ST10, where steelmaking dust was used as an additive, there was a 5.7-fold increase in crushing resistance (CS = 11.1 MPa) and a reduction in water absorption to 11.5%. Water absorption was high in the other tests, exceeding 32%. In summary, the strength test results indicated that the obtained aggregate strength was significantly greater than that of most aggregates available on the market.

The crushing strengths of various commercial aggregates are as follows: Liapor: 0.7–10 MPa; Arlita: 0.98 MPa; Lytag: 0.43 MPa; LECA and Ardelite: 0.09 MPa; and LECA Gniew: 0.7–4.0 MPa [58,59,60]. Compared to the results published for other wastes used to produce geopolymer granules, the obtained values of CS amounted 6.7 MPa (CFA + CR) and 11.1 MPa (CFA + SD) are among the hardest for materials cured under ambient conditions with a limited amount of activator. It should be emphasized that although the strength of the granules obtained (especially ST10) is close to the parameters of some dense (natural) aggregates, the main reference group for us remains manufactured lightweight aggregates (LWA). This is due to the fact that our cold bonding technology is a direct, low-energy alternative to the energy-intensive sintering processes used in the production of LWA. In addition, the high water absorption of most of our granules (above 32%) confirms their porous structure, typical of lightweight aggregates, and fundamentally different from the almost zero water absorption of dense aggregates. Our material therefore fills a market niche, offering strength significantly superior to typical LWAs, while maintaining a low-energy production regime.

### 3.4. Chemical and Mineral Composition of the Geopolymer-Based Granulates

Table 8 and Table 9 present the chemical and mineral compositions of the geopolymer-based granulates after 28 days of curing. Table 10 presents the results of selected metal leachability from the tested granulates. Samples ST2 and ST3 (coal fly ash-based) were dominated by typical silicate minerals and calcium phases such as quartz, feldspar, portlandite, anhydrite and muscovite, and exhibited a balance between the contents of amorphous and crystalline substances. The ratios of the amorphous to crystalline contents were 0.94 and 1.02 for samples ST2 and ST3, respectively. In samples obtained on the basis of the coal fly ash (CFA) and composite regrind (CR), a gradual decrease in quartz content from 31.5 wt% (content in the CFA) to 10.5 wt% (content in sample ST7) and an increase in the amorphous content from 35.5 wt% (content in CFA) to 70.0 wt% (content in ST7) was observed.

The ratios of the amorphous to crystalline contents were 2.09 and 2.33 for samples ST5 and ST7, respectively. This significant increase in the amorphous-to-crystalline ratio (A:C) in samples ST5 and ST7 provides direct mineralogical evidence of a higher degree of substrate reaction and a more effective geopolymerization process. This suggests that the CR additive did not act merely as an inert filler, but may have actively participated in the reaction or facilitated the dissolution of aluminosilicates from fly ash, leading to the precipitation of a larger volume of amorphous geopolymer binder. It is this amorphous phase that is the main carrier of mechanical properties, which directly explains the significant increase in the strength of these granules observed in Section 3.3.

Sample ST10 (based on coal fly ash and steelmaking dust) exhibited a different mineral composition than the other geopolymer samples. The main components included zinc and iron minerals, i.e., franklinite (32.0 wt%), zincite (15.0 wt%), magnetite (9.0 wt%) and hematite (2.0 wt%). Quartz accounted for 5.5 wt%, while the amorphous phase accounted for 25.5 wt%. The ratio of amorphous to crystalline content was 0.35, therefore crystalline phases dominated in this material, as in the steelmaking dust (SD).

**Figure 10:**
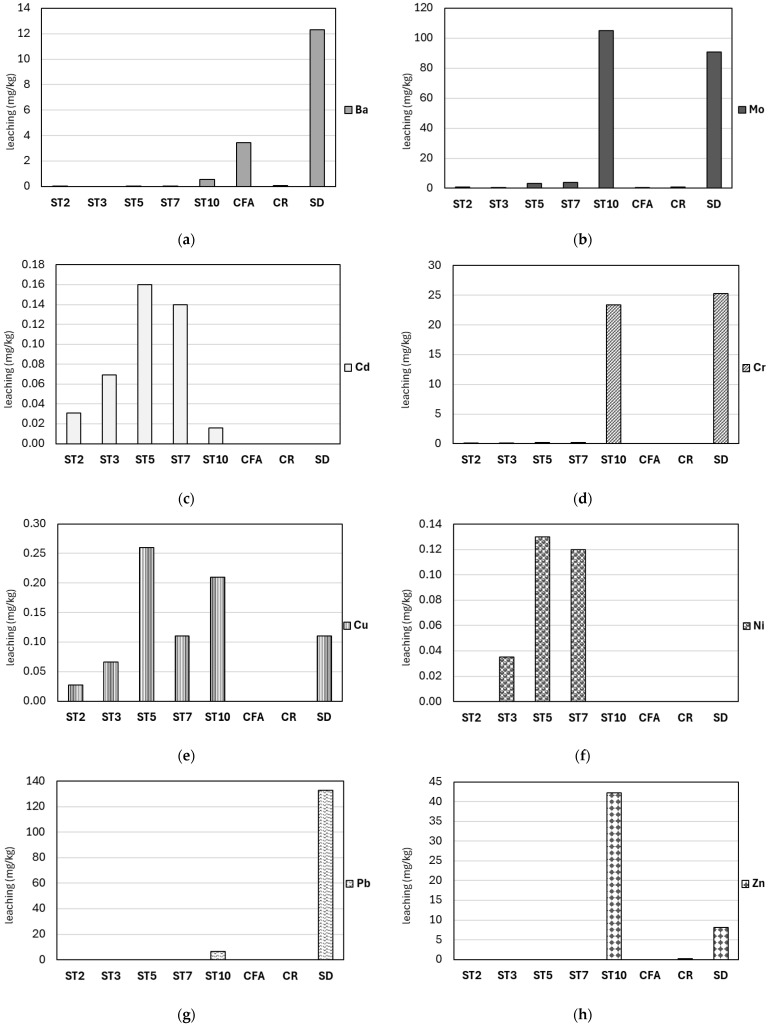

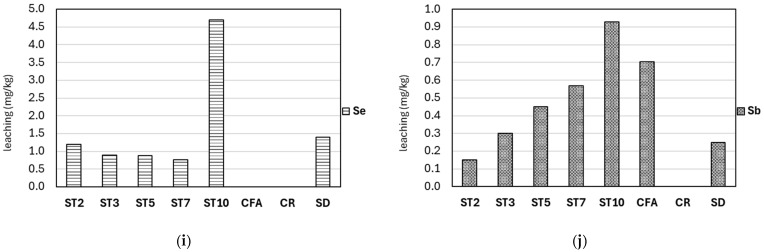
Leaching content of selected metals: (**a**) barium, (**b**) molybdenum, (**c**) cadmium, (**d**) chromium, (**e**) copper, (**f**) nickel, (**g**) lead, (**h**) zinc, (**i**) selenium, (**j**) antimony from the input materials (CFA, CR, SD) and the geopolymer-based granulate (ST2, ST3, ST5, ST7, ST10); Note: Explanations of abbreviations are provided in Table 2.

Figure 10 compares the leachability of selected metals from the input materials and the granules obtained on their basis. These values were compared with the guidelines for the acceptance of waste for disposal in inert waste landfills [61]. Exceeded permissible leaching limits were observed in the following ranges:

- geopolymer granules based on coal fly ash (ST3, ST2)—the permissible leaching limits were exceeded for cadmium (only in ST3), selenium, molybdenum and antimony;
- geopolymer granules based on coal fly ash and composite regrind (ST5 and ST7)—the permissible leaching limits were exceeded for cadmium, selenium, molybdenum and antimony;
- geopolymer granules based on coal fly ash and steelmaking dust (ST10)—the permissible leaching limits for most metals were exceeded: chromium, lead, zinc, selenium, molybdenum and antimony.

Alkaline activators, which increase leachate pH, were used in the geopolymer synthesis. A change in pH has a significant effect on metal immobilization [45]. The following trends were observed: (i) barium, antimony, lead—higher leachability of these metals from the input materials than the geopolymer-based granules; (ii) molybdenum, cadmium, chromium, copper, nickel, zinc, selenium—higher leachability of these metals from the geopolymer-based granules than the input materials.

As pH decreases, metals are released from soil particles due to reduced adsorption and increased dissolution, especially for metals like cadmium and zinc, which are highly mobile even at neutral or slightly alkaline pH. Some metals (notably Pb and Zn) show amphoteric behavior: their leaching is high at both low and high pH, but minimized at intermediate pH values. The effect of pH on metal leaching is also influenced by material properties (buffer capacity, organic matter, mineral composition) and the specific metal involved.

The leachability of chromium, zinc, and molybdenum is significantly higher with CFA + SD geopolymer granules (ST10) than with the input materials. The leachability of lead from sample ST10 is lower than from its synthesis precursors. The pH of leachate from steelmaking dust was 12.40, while the pH of the leachate from ST10 granulates was 12.60, so the alkaline activators used may have a key influence on the mobilization/immobilization of metals.

NaOH can selectively leach zinc from franklinite, especially at high concentrations and elevated temperatures, while iron remains largely insoluble. This allows for efficient zinc recovery with minimal iron dissolution. ZnO is highly soluble in NaOH, forming soluble zincate ions ([Zn(OH)_4_]^2−^). The leaching process is chemically controlled and efficient, with high zinc recovery rates [62]

NaOH, especially when combined with oxidants or at high temperatures, efficiently extracts chromium from chromite and spinel phases as water-soluble chromate. The process is enhanced by oxidizing agents (e.g., H_2_O_2_) and high alkali concentrations [63]. NaOH is very effective for leaching molybdenum from minerals like wulfenite, with extraction rates above 99% at elevated temperatures and high alkali concentrations [64]. NaOH is highly effective for leaching lead from oxide and some secondary minerals, under optimal conditions (high temperature, high NaOH concentration). However, in some matrices (e.g., fly ash), NaOH can also stabilize lead, reducing its leachability by forming less soluble compounds [65].

The observed high leachability of Cr, Zn, and Mo from ST10 granules is not only a technical disqualification of this variant, but also a key scientific discovery. It demonstrates the fundamental duality and challenge of alkaline activation of metallurgical waste. The strongly alkaline environment necessary for the dissolution of aluminosilicates is also an aggressive chemical agent. As already mentioned, these conditions selectively attack the mineral phases present in SD, such as franklinite and zincite, leading to the mobilization of heavy metals. This illustrates a fundamental process relationship: conditions optimal for geopolymerization (high pH) may also be conditions conducive to the leaching of amphoteric contaminants (Pb, Zn) or those that form soluble oxyanions (Cr, Mo).

### 3.5. Geopolymer-Based Granulate Surface Morphology

**Figure 11:**
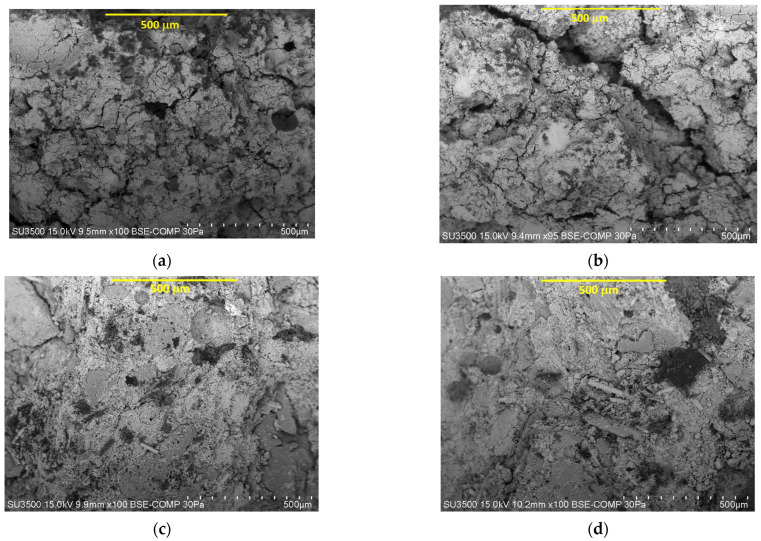
Surface area comparison for the CFA geopolymer granulate (represented by sample ST3, (**a**,**b**)) and CFA + CR geopolymer granulate (represented by sample ST7, (**c**,**d**)).

**Figure 12:**
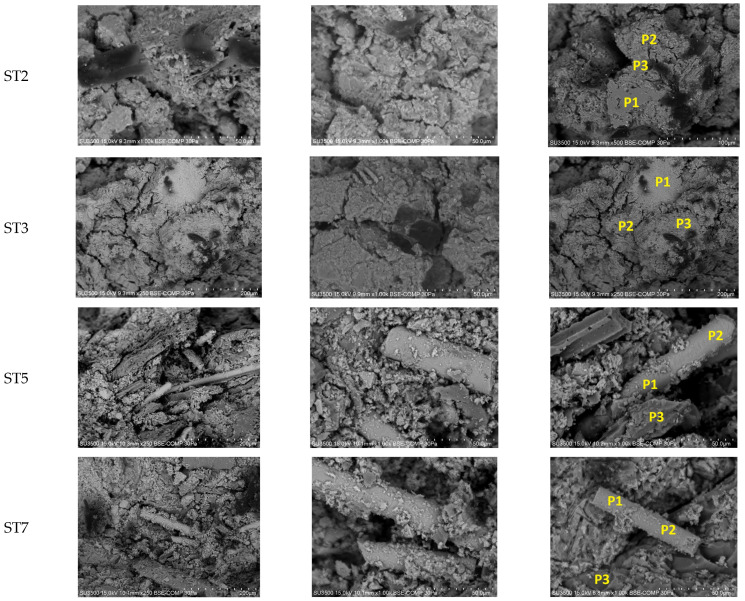
SEM images of selected geopolymer-based granulates ST2, ST3, ST5, ST7; Note: Explanations of abbreviations are provided in Table 2.

Figure 11 and Figure 12 present SEM images of geopolymer granules based on coal fly ash (CFA) as well as geopolymer granules based on coal fly ash and composite regrind (CFA + CR) after 28 days of curing. The chemical composition of the selected micro-areas corresponding to Figure 12 is presented in Table 11.

Pławecka et al. [45] assessed whether mechanically ground waste from decommissioned wind turbine blades can be used as a filler (0%, 5%, 15%, 30% by dry mass) in fly ash-based geopolymer composites to reduce waste and substitute conventional fillers. The compressive strength of the materials with waste fraction additions was lower than that of the reference sample. Low-to-moderate additions (around 5–15%) can yield materials with acceptable mechanical properties. Studies have demonstrated that the main drawback was high porosity and that reducing the porosity (improved mixing, vibration or modified processing) could improve strength.

This study has demonstrated that the structure of the CFA + CR geopolymer granules (based on fly ash and composite regrind) (Figure 11c,d) was more compact and dense; the pores were smaller, and the material was better bound than the CFA geopolymer granules (based on fly ash) (Figure 11a,b). The CFA + CR geopolymer structure was characterized by the presence of fibers whose lengths ranged from a few micrometers up to several hundred micrometers. An EDS analysis indicated that the main components of the fibers included silicon, calcium and aluminum. The presence of titanium, sodium and magnesium was identified as well (Table 11). This analysis showed that the presence of composite regrind (Si–Ca–Al) fibbers promotes the formation of structural bridges and surface sealing, which confirms the observed increase in strength and decrease in water absorption compared to ash granules alone. Microstructure analysis (Figure 11 and Figure 12) provides an explanation for the strength increase mechanism observed in Section 3.3. While the base CFA granulate (Figure 11a,b) exhibits a porous structure with numerous voids and loosely bound grains, the structure of the CFA + CR granulate (Figure 11c,d) is fundamentally different. It is clearly more compact and dense, and, crucially, permeated with fibers from the composite milling. As shown by EDS analysis (Table 11, e.g., areas P1, P2 for ST5 and ST7), these fibers (with a Si-Ca-Al composition) are not a passive filler, but are well integrated into the amorphous geopolymer matrix. They act as micro-reinforcement and “structural bridges,” transferring stresses and hindering the propagation of microcracks. This direct reinforcement at the micrometric level is the scientific reason for the observed more than 3-fold increase in compressive strength (up to 6.7 MPa).

Research on geopolymers synthesized from coal fly ash highlights the critical influence of microstructure on the final mechanical properties, durability and environmental performance [66]. This study also showed a clear correlation between the structure of the geopolymer and the mechanical properties of the granules. In this case, the structural cohesion was enhanced by the use of an intensive counter-current mixer for granulate production combined with the presence of fibers.

### 3.6. Justification of Potential as a ‘Sustainable Substitute’

The research results confirm that the developed geopolymer granules, especially those with PK added, are a viable and sustainable substitute for natural aggregates.

The substantiation of this thesis is based on proven facts. First, the ‘sustainable’ aspect results from a triple benefit: (1) the valorization of three different industrial waste streams (PM, PK, PS), which is in line with the principles of the circular economy and responds to the challenges described in the Introduction; (2) the use of energy-efficient ‘cold bonding’ technology, which allows curing at room temperature; and (3) demonstrated in the process of optimization and reduction in alkaline activator consumption (S/L > 2.0).

Secondly, the ‘substitute’ aspect has been clearly confirmed by mechanical properties. It has been shown that the addition of PK or PS leads to granules with crush strength (>6 MPa and >11 MPa, respectively) that ‘surpasses many commercial lightweight aggregates’. Thus, it has been proven that these materials are not an inferior substitute, but a fully fledged, and in terms of strength often superior, technical product capable of replacing natural aggregates in demanding applications.

### 3.7. Economic Feasibility and Process Cost Implications

The developed method for producing geopolymer granules was designed with economic factors in mind. Unlike traditional lightweight sintered aggregates, the proposed solution is based on a granulation and curing process at room temperature (‘cold bonding’). Eliminating the sintering stage is crucial for profitability. As indicated in the literature, the production of aggregates using the ‘cold bonding’ method consumes less energy and is more cost-effective than sintering, which translates into lower operating costs.

An additional factor reducing process costs is the achieved high solid-to-liquid ratio (S/L > 2.0). As shown in the Conclusions, this has enabled a significantly reduced amount of costly alkaline activators (NaOH and Na_2_SiO_3_). Lower consumption of activators, which have been a frequent cost factor in geopolymerization processes, is a direct result of the use of an intensive counter-rotating mixer.

From an economic point of view, the main advantage of the proposed solution is the use of waste raw materials (fly ash, steel mill dust, and composite grinding). As indicated in the Introduction, research on the recycling and reuse of waste materials is crucial for sustainable development. The use of these materials as feedstock minimizes their acquisition costs and is in line with the circular economy strategy.

It has been demonstrated that the production process can be carried out in a single technological operation, using an intensive counter-rotating mixer and an ambient maturation system. Low energy consumption (resulting from cold bonding) and limited demand for activators (resulting from high S/L) make this technology a promising industrial alternative.

In direct comparison with natural aggregates, whose resources are rapidly depleting, the unit cost of producing geopolymer granules may be higher. However, the overall profitability of the process is supported by the elimination of energy costs (compared to sintering) and reduced activator costs (thanks to S/L > 2.0). From an economic point of view, the proposed process therefore represents a viable alternative, particularly in the context of industrial waste valorization.

### 3.8. Scope of Environmental Analysis and Context of Alkaline Activators

It should be emphasized that the scope of the environmental analysis in this study focused on the valorization of industrial waste (PM, PK, PS), the energy efficiency of the cold bonding process compared to sintering, and the assessment of the washability of contaminants from the final product. The issue of quantitative environmental impact assessment (LCA) related to the production of alkaline activators (NaOH and Na_2_SiO_3_) itself was beyond the scope of this study. However, this issue was addressed indirectly through process optimization. It was demonstrated that the developed technology, thanks to the use of an intensive counter-rotating mixer, allows operation at a high solid-to-liquid ratio (S/L > 2.0). As indicated in the Conclusions, this results in a “significantly reduced amount” of alkaline activators consumed. The reduction in the consumption of these components is crucial because it directly minimizes the overall environmental footprint associated with their production, while improving the economic profitability of the process.

### 3.9. Summary

The developed method of granulation and cold bonding allowed for obtaining granules with compressive strength exceeding 6 MPa (CFA + CR) and 11 MPa (CFA +SD), which places them above the range of typical values for commercial lightweight aggregates. These parameters confirm that the proposed material meets the technical criteria for use as an aggregate in structural concrete or prefabricated elements. These studies have shown that the addition of composite grinding (CR) fundamentally modifies the geopolymerization process. This leads to the formation of a compact, fiber-reinforced microstructure, as confirmed by SEM analyses. This explains the mechanism by which above-average compressive strength (>6 MPa) was achieved, higher than that of many commercial lightweight aggregates (such as LECA, Liapor, Lytag). At the same time, the addition of steel dust (SD) resulted in the highest strength (>11 MPa), which is related to the different mineral composition of the final product.

The results indicate also that CFA + SD granules (ST10), despite their highest mechanical strength (11.1 MPa), exceed the permissible values for Cr, Pb, and Mo leaching, which limits their direct use or storage as inert materials. Therefore, this variant should be treated as a material for use in closed structures, e.g., in cement or geopolymer composites, where further binding reactions can ensure more effective immobilization of heavy metals.

In order to improve chemical stability, it is proposed to modify the alkaline activator (increase the silicate modulus, partial replacement of Na with K), introduce pozzolanic additives (metakaolin or GGBS—Ground Granulated Blast Furnace Slag) or apply surface protective coatings of a thin layer of geopolymer or cement grout. Therefore the key scientific conclusion is the identification of the dual role of steel dust (SD) additive. While it significantly improves mechanical parameters, a fundamental material challenge has been identified: the strongly alkaline conditions required for activation also mobilize hazardous heavy metals (Cr, Pb, Mo), leading to exceedances of standards. This points to the need for further research on immobilization in geopolymer matrices based on steel mill waste.

Another important innovative aspect of the developed technology is the high solid-to-liquid ratio (S:L > 2.0), which has made it possible to reduce the consumption of alkaline activators (NaOH and Na_2_SiO_3_) in the synthesis process. The reduction in the alkaline liquid content by over 30% compared to standard recipes translates directly into lower production costs and carbon footprint, confirming the practical industrial potential of the process.

Due to their favorable mechanical properties and volume stability, these granulates have the potential be used both as lightweight aggregate for concrete and precast elements, as well as ballast or insulation material in road and civil engineering. The use of industrial waste as the primary raw material makes the developed method an economically and environmentally sustainable solution.

## 4. Conclusions

The conclusions, which are based on the mechanical and physicochemical analysis results for geopolymer-based granulates with a substitution of coal fly ash (CFA) by composite regrind (CR) or steelmaking dust (SD), are as follows:

(1) The use of an intensive counter-current mixer enabled the mixing and granulation of the feedstock in the form of fluidized fly ash, alkaline activators, and additives such as composite regrind or steelmaking dust in a single technological operation. Moistening and/or powdering the ash-based granule surfaces had a significant effect on the quality of the obtained product, i.e., the size and shape of the geopolymer granules.
(2) The developed method enabled the production of geopolymer granules with a significantly reduced amount of water glass and the NaOH solution (solid-to-liquid ratio above 2.0).
(3) The produced geopolymer-based granulates had a compact, stable structure and were capable of self-hardening at room temperature without the need for further processing. The crushing resistance of the CFA + CR (>6 MPa) and CFA + SD (>11 MPa) geopolymer granules was greater than that of most aggregates available on the market.
(4) The method used to produce the CFA + CR geopolymer granules ensured the stabilization of metals such as chromium, copper, nickel, zinc, lead and barium. In the case of the CFA + SD geopolymer granules, the leaching levels of metals such as chromium, zinc and molybdenum were exceeded several times relative to the permissible leaching limits for the acceptance of waste for disposal in inert waste landfills.
(5) Considering both the mechanical and chemical properties of the produced geopolymer granules, the use of composite regrind as an additive is promising. In the next stage of the work, the geopolymer granule production process will be optimized to improve the immobilization of metals such as cadmium, selenium, molybdenum and antimony.

Taking into account both strength and environmental parameters, the CR additive should be considered the most promising component for cold-cured geopolymer granules. Despite its high strength, the DS additive requires additional metal stabilization methods to meet the environmental requirements for inert waste landfill.

The results obtained clearly confirm that geopolymer granules produced under cold curing conditions are a viable alternative to natural aggregates. The developed technology is characterized by low energy consumption, limited use of activators, and the possibility of using a wide range of industrial waste as raw materials. Combined with mechanical properties that exceed those of commercially available lightweight aggregates, these materials have high implementation potential in the construction sector, contributing to the reduction in natural resource exploitation.

From an economic point of view, the developed technology has key advantages for its potential profitability. The process is characterized by low energy consumption, as it is based on an energy-efficient cold bonding method and granule curing at room temperature. At the same time, significantly reduced consumption of expensive alkaline activators (NaOH solution and water glass) has been demonstrated thanks to the high solid-to-liquid ratio (S/L > 2.0) achieved in the process. Raw material costs are minimized by using industrial waste (CFA, CR, DS). The combination of these factors—energy efficiency, reduced consumption of activators, and the use of waste raw materials—makes the proposed technology promising and potentially competitive.

The authors acknowledge that while the term ‘energy-efficient’ is used in this paper to describe the underlying principles of the geopolymerization process (i.e., low-temperature processing and the valorization of industrial by-products which would otherwise require disposal), this study does not contain a quantitative, comparative evaluation of the energy consumption of the proposed technology versus the conventional extraction and processing of natural aggregates. Natural aggregate production, while mechanically intensive, often involves lower inherent energy input compared to manufacturing processes.

Therefore, to definitively validate the ‘energy-efficient’ and ‘sustainable’ nature of these geopolymer-based granulates in the context of substituting natural aggregates, a comprehensive Life Cycle Assessment (LCA) is required. Such an analysis was beyond the scope of this initial technological study but remains a critical objective for future research. This future work must quantify the energy inputs and environmental impacts across the entire value chain—from raw material extraction (for activators) and transport, through processing, to the end-of-life considerations of the final product—and compare these metrics directly against those for natural aggregates.

## 5. Patents

Patent application (Poland), “Method for Producing Geopolymers Using an Intensive Counter-Current Mixer”/“Sposób Wytwarzania geopolimerów z wykorzystaniem intensywnego mieszalnika przeciwbieżnego” (PW 6/2025).

## Figures and Tables

**Table 1:** Research on the physical and mechanical properties of geopolymer-based artificial aggregates [32].

References	Artificial Aggregates Produced	Physical Properties			Mechanical Properties		
		Specific Gravity	Density, kg/m^3^	Water Absorption, %	AIV, %	ACV, %	CS, MPa
[33]	FA-RM	n.d.	1007–1132	9.80–12.10	n.d.	n.d.	1.46–6.18
[34]	FA-SF	1.700	738	18.98	10.24	n.d.	2.03–12.00
[35]	FA-GGBS	n.d.	764–878	18.73–28.30	25.00–39.00	n.d.	n.d.
[36]	FA-SF	1.800	710	17.95	10.03	n.d.	3.34–4.54
[37]	FA-BT, FA-MK, FA-GGBS	1.68–1.89	848–983	13.01–21.26	31.96–50.47	n.d.	14.51–22.81
[38]	FA	n.d.	1450–1500	22.00–23.00	n.d.	n.d.	n.d.
[39]	FA	2.058	n.d.	7.07	28.31	23.96	n.d.
[40]	CFA, FFA	2.40–2.45	n.d.	5.51–6.05	n.d.	n.d.	n.d.

Notes: AIV—Aggregate Impact Value; ACV—Aggregate Crushing Value; CFA—Class C fly ash; FFA—Class F fly ash; FA—fly ash; GGBS—ground granulated blast furnace slag; MK—metakaolin; n.d.—no data; RM—red mud; SC—Crushing Strength; SF—silica fume.

**Table 2:** Mixture proportions for forming geopolymer-based granulates.

Exp. Index	Solid: Liquid	Solid Compound	CFA:SD or CFA:CR	NaOH:Na_2_SiO_3_	M	P
ST1	1.50	CFA	-	3.0	no	no
ST2A	2.00	CFA	-	3.0	no	no
ST2	1.90	CFA	-	3.0	yes	no
ST3	1.75	CFA	-	3.0	no	yes
ST5	1.55	CFA + CR	0.67	3.0	yes	no
ST7	1.96	CFA + CR	0.50	3.0	yes	yes
ST10	2.54	CFA + SD	0.40	3.0	no	no

Note: CFA—coal fly ash, CR—composite regrind, SD—steelmaking dust, M—moisturizing, P—powdering, Solid = CFA + CR + SD + P, Liquid = NaOH + Na_2_SiO_3_ + M.

**Table 3:** Main chemical composition (via XRF) of the materials used for the geopolymer-based granulate production.

Sample Index	CaO	SiO_2_	Al_2_O_3_	Fe_2_O_3_	MgO	Na_2_O	K_2_O	TiO_2_	ZnO	MnO	P_2_O_5_	SO_3_	Cl	Br	C	H
	wt%															
CFA	20.90	30.00	22.20	5.39	1.93	2.71	2.06	1.09	0.04	0.09	0.16	8.25	0.77	0.01	4.08	<0.01
CR	19.60	30.70	9.10	0.59	1.14	0.32	0.30	1.06	<0.01	0.02	0.04	0.02	0.02	<0.01	34.10	2.88
SD	8.85	2.09	0.73	37.20	2.46	<0.01	1.37	0.06	36.20	2.46	0.13	1.42	1.80	0.03	2.62	<0.01

**Table 4:** Trace chemical composition (via ICP–OES) of the materials used for the geopolymer-based granulate production.

Sample Index	As	Ba	Cd	Co	Cr	Cu	Mo	Ni	Pb	Sb	Se	Sn	V	Sr	Li	W
CFA	0.33	317	2.17	10.8	67.1	64.6	1.56	37.2	175	3.09	2.04	2.25	94.1	245	82.8	<4
CR	0.29	33.8	<1	81.0	16.1	5.54	10.5	7.90	1.32	<1	3.16	9.31	7.88	35.2	22.2	<4
SD	<0.1	236	341	37.0	3604	1142	272	1031	9518	118	17.4	181	59.6	68.0	20.1	14.2

**Table 5:** Water leaching results (via ICP–OES) for selected elements from the geopolymer-based granulate (expressed as mg/kg of solid dry material) and pH of the water leachate.

Sample Index	Mn	Cd	Co	Cr	Cu	Ni	Pb	Zn	As	Se	Mo	V	Ti	Sb	Ba	Sr	Li	pH
	mg/kg s.d.m.																	-
CFA	<0.002	<0.005	<0.01	<0.05	<0.01	<0.02	<0.05	<0.05	<0.05	<0.05	0.509	<0.05	<0.005	0.703	3.46	53.7	1.34	12.58
CR	0.461	<0.005	2.35	<0.05	<0.01	<0.02	<0.05	0.224	<0.05	<0.05	0.854	<0.05	<0.005	<0.05	0.08	0.31	0.311	7.68
SD	<0.002	<0.005	<0.01	25.3	0.11	<0.02	133	8.10	<0.05	1.40	90.9	<0.05	0.053	0.25	12.3	11.1	3.02	12.40

**Table 6:** Particle size (number distribution) of the materials used for the geopolymer-based granulate production.

CE Diameter	CFA	CR	SD
µm	%		
<5	84.7	68.0	93.7
5–10	11.5	16.4	5.87
10–20	3.18	10.51	0.65
20–50	0.632	4.71	0.11
50–100	0.020	0.312	0.004
100–200	0.001	0.034	<0.001
>200	n.d.	0.008	<0.001

Note: n.d.—not detected.

**Table 7:** Particle size (volume distribution) of the materials used for the geopolymer-based granulate production.

CE Diameter	CFA	CR	SD
µm	%		
<5	4.48	0.19	12.3
5–10	11.0	1.14	13.6
10–20	23.7	5.21	13.7
20–50	41.3	21.5	18.9
50–100	13.9	13.8	8.53
100–200	5.64	13.5	5.44
200–400	n.d.	26.3	27.6
>400	n.d.	18.4	n.d.

Note: n.d.—not detected.

**Table 8:** Mineral composition of the geopolymer-based granulate.

Phase	Sample Index				
	ST2	ST3	ST5	ST7	ST10
	wt%				
Quartz (SiO_2_)	26.5	22.5	12.5	10.5	5.5
Franklinite (ZnFe_2_O_4_)	n.d.	n.d.	n.d.	n.d.	32.0
Zincite (ZnO)	n.d.	n.d.	n.d.	n.d.	15.0
Calcite (CaCO_3_)	5.5	5.5	3.5	3.5	4.5
Kaolinite (Al4[Si_4_O_10_(OH)_8_])	n.d.	n.d.	n.d.	n.d.	n.d.
Feldspars (potassium + plagioclases)	5.0	4.0	2.0	4.0	1.0
Anhydrite (CaSO_4_)	4.0	4.0	5.0	4.0	4.0
Portlandite (Ca(OH)_2_)	3.0	3.0	2.0	2.0	n.d.
Hematite (Fe_2_O_3_)	2.0	2.0	2.0	2.0	2.0
Apatite (Ca[(F/OH/Cl/(PO_4_)_3_)	2.0	3.0	4.0	2.0	n.d.
CaO substance	1.0	1.0	1.0	2.0	n.d.
Muscovite (KAl_2_[AlSi_3_O_10_(OH)_2_])	1.0	2.0	n.d.	n.d.	n.d.
Magnetite (FeFe_2_O_4_)	1.0	2.0	n.d.	n.d.	9.0
Chlorite ((Mg,Fe)_6_[(AlSi_3_)O_10_](OH)_8_)	n.d.	n.d.	n.d.	n.d.	n.d.
Crystalline substance (C)	51.0	49.0	32.0	30.0	73.0
Amorphous substance (A)	48.0	50.0	67.0	70.0	25.5
A:C phase ratio	0.94	1.02	2.09	2.33	0.35

Note: n.d.—not detected.

**Table 9:** Main chemical composition (via XRF) of the geopolymer-based granulate.

Sample	CaO	SiO_2_	Al_2_O_3_	Fe_2_O_3_	MgO	Na_2_O	K_2_O	TiO_2_	ZnO	MnO	P_2_O_5_	SO_3_	Cl	Br	C	H
	wt%															
ST2	30.10	22.60	10.70	9.60	0.83	11.40	2.08	1.48	0.11	0.09	0.11	5.87	0.63	0.02	3.42	0.55
ST3	30.20	22.00	9.36	10.30	0.70	13.20	1.89	1.37	0.12	0.12	0.13	5.40	0.64	0.02	3.66	0.51
ST5	18.60	29.50	9.14	2.89	0.91	20.20	0.91	0.79	0.03	0.03	0.06	2.20	0.33	0.00	14.30	<0.01
ST7	19.70	28.00	8.11	2.81	0.80	17.70	0.94	0.98	0.03	0.04	0.08	1.60	0.31	0.01	17.30	1.38
ST10	14.10	11.80	4.12	27.20	1.89	4.20	1.42	0.28	24.40	1.65	0.31	2.42	1.28	0.03	3.22	<0.01

**Table 10:** Water leaching results (via ICP–OES) of selected elements from the geopolymer-based granulate (expressed as mg/kg of solid dry material) and pH of the water leachate.

Sample	Mn	Cd	Co	Cr	Cu	Ni	Pb	Zn	Se	Mo	Sn	V	Ti	Sb	Ba	Sr	Li	W	pH
	mg/kg s.d.m.																		-
ST2	0.003	0.031	<0.01	0.16	0.027	<0.02	<0.05	<0.05	1.2	0.83	0.056	12.3	0.019	0.15	0.005	0.13	11.5	1.87	12.84
ST3	0.013	0.069	<0.01	0.17	0.066	0.035	<0.05	<0.05	0.9	0.63	<0.05	9.4	0.010	0.3	<0.002	0.16	8.9	1.18	12.74
ST5	0.035	0.16	0.29	0.21	0.26	0.13	<0.05	<0.05	0.88	3.2	0.097	10.6	0.023	0.45	0.041	0.26	0.27	1.27	11.73
ST7	0.0029	0.14	0.23	0.2	0.11	0.12	<0.05	<0.05	0.76	3.8	<0.05	9.1	0.019	0.57	0.036	0.2	2.1	1.64	11.31
ST10	<0.002	0.016	<0.01	23.4	0.21	<0.02	6.4	42.2	4.7	105	0.12	8.7	<0.005	0.93	0.56	0.16	3.3	12.3	12.60
Limit ^1^	nd	0.04	nd	0.50	2.00	0.40	0.50	4.00	0.10	0.50	nd	nd	nd	0.06	20.00	nd	nd	nd	-

Note: ^1^ Permissible leaching limit values for the acceptance of waste at inert waste landfills [61].

**Table 11:** Chemical composition (via EDS) of selected micro-areas of the geopolymer-based granulate.

Sample Index ^1^	Micro-Area Index ^2^	C	O	Na	Mg	Al	Si	K	Ca	Ti	Fe
		wt%									
ST2	P1	5.1	42.8	1.9	-	9.9	30.3	10.2	-	-	-
	P2	6.5	40.5	8.1	0.8	11.5	20.8	1.5	7.4	-	2.9
	P3	31.4	31.6	7.6	0.5	6.6	13.3	1.0	6.6	-	1.6
ST3	P1	3.8	38.9	1.8	-	1.8	3.8	-	48.7	-	1.2
	P2	4.1	41.2	9.9	0.5	8.7	16.5	0.9	18.2	-	-
	P3	3.8	44.5	3.7	1.3	9.9	15.0	0.4	18.6	0.9	2.0
ST5	P1	9.2	42.7	1.5	0.7	7.0	25.5	-	13.6	-	-
	P2	8.4	42.0	1.1	0.6	7.3	25.6	-	14.5	0.5	-
	P3	47.7	33.1	2.8	-	2.7	8.5	0.6	4.7		-
ST7	P1	6.1	41.5	2.0	0.6	7.2	27.3	-	14.8	0.6	-
	P2	8.8	37.3	1.2	0.5	7.6	27.4	-	15.7	0.7	1.0
	P3	54.6	33.4	1.6	-	1.5	5.2	-	3.7	-	-

Note: ^1^ Explanations of abbreviations are provided in Table 2; ^2^ The micro-areas (P1, P2, P3) are marked in Figure 12.

## Data Availability

The original contributions presented in this study are included in the article. Further inquiries can be directed to the corresponding authors.

## Abbreviations

The following abbreviations are used in this manuscript:

AIV	Aggregate Impact Value
ACV	Aggregate Crushing Value
CFA	Class C fly ash
CE	Circle Equivalent diameter
FFA	Class F fly ash
FA	Fly Ash
GGBS	ground granulated blast furnace slag
MK	Metakaolin
RM	Red Mud
CFA	Coal fly ash from the combustion of hard coal in a fluidized coal boiler (material used in the experimental section)
CR	Composite regrind (material used in the experimental section)
DS	Steelmaking dust (material used in the experimental section)
CS	Crushing Strength
SF	Silica Fume
Na_2_SiO_3_	Sodium Silicate
NaOH	Calcium Hydroxide
XRF	X-ray Fluorescence
XRD	dispersive X-ray fluorescence
